# Analysis of the Impact of Detection Threshold Adjustments and Noise Uncertainty on Energy Detection Performance in MIMO-OFDM Cognitive Radio Systems

**DOI:** 10.3390/s22020631

**Published:** 2022-01-14

**Authors:** Josip Lorincz, Ivana Ramljak, Dinko Begušić

**Affiliations:** 1 Faculty of Electrical Engineering, Mechanical Engineering and Naval Architecture (FESB), University of Split, R. Boskovica 32, 21000 Split, Croatia; dinko.begusic@fesb.hr; 2 Elektroprenos—Elektroprijenos BiH” a.d. Banja Luka, 88000 Mostar, Bosnia and Herzegovina; ivana.ramljak@elprenos.ba

**Keywords:** spectrum sensing, energy detection, SLC, OFDM, noise uncertainty, dynamic threshold, MIMO, SISO, cognitive networks, SNR, probability, wireless, false alarm, transmit, receive, power

## Abstract

Due to the capability of the effective usage of the radio frequency spectrum, a concept known as cognitive radio has undergone a broad exploitation in real implementations. Spectrum sensing as a core function of the cognitive radio enables secondary users to monitor the frequency band of primary users and its exploitation in periods of availability. In this work, the efficiency of spectrum sensing performed with the energy detection method realized through the square-law combining of the received signals at secondary users has been analyzed. Performance evaluation of the energy detection method was done for the wireless system in which signal transmission is based on Multiple-Input Multiple-Output—Orthogonal Frequency Division Multiplexing. Although such transmission brings different advantages to wireless communication systems, the impact of noise variations known as noise uncertainty and the inability of selecting an optimal signal level threshold for deciding upon the presence of the primary user signal can compromise the sensing precision of the energy detection method. Since the energy detection may be enhanced by dynamic detection threshold adjustments, this manuscript analyses the influence of detection threshold adjustments and noise uncertainty on the performance of the energy detection spectrum sensing method in single-cell cognitive radio systems. For the evaluation of an energy detection method based on the square-law combining technique, the mathematical expressions of the main performance parameters used for the assessment of spectrum sensing efficiency have been derived. The developed expressions were further assessed by executing the algorithm that enabled the simulation of the energy detection method based on the square-law combining technique in Multiple-Input Multiple-Output—Orthogonal Frequency Division Multiplexing cognitive radio systems. The obtained simulation results provide insights into how different levels of detection threshold adjustments and noise uncertainty affect the probability of detection of primary user signals. It is shown that higher signal-to-noise-ratios, the transmitting powers of primary user, the number of primary user transmitting and the secondary user receiving antennas, the number of sampling points and the false alarm probabilities improve detection probability. The presented analyses establish the basis for understanding the energy detection operation through the possibility of exploiting the different combinations of operating parameters which can contribute to the improvement of spectrum sensing efficiency of the energy detection method.

## 1. Introduction

The increased popularity of wireless communication networks raises the need for an improvement of network capacity and the efficiency of spectrum usage. To address the problem of the efficient usage of the spectrum, the cognitive radio networks (CRN) concept was proposed as a promising solution that can be implemented in wireless communication systems. In CRNs, two types of users known as the primary users (PUs) and the secondary users (SUs) are known. The cognitive radio (CR) enables SU to perform dynamic spectrums access (DSA) in periods when PU does not use the spectrum. This means that the PU always has the priority when exploiting a dedicated licensed spectrum. SU may use licensed bands so long as it does not cause interference with PU. Therefore, the main goal of DSA in CRNs is to improve the efficiency of the spectrum usage [1,2]. 

Spectrum sensing (SS), as an essential function of CRN, enables users in cognitive networks to have information about its environment and spectrum availability. The most widely used SS method is energy detection (ED). The ED method is a local non-cooperative SS method that does not demand prior knowledge about the characteristics of the PU signal. Compared to other prominent local SS methods, ED has the least computational and implementation complexity [3,4]. However, the ED is very sensitive to fluctuations in noise power, low values of the signal-to-noise ratio (SNR), and fading [5]. Therefore, the performance of ED is confined by the noise power variations which is also defined as noise uncertainty (NU). In real wireless communication systems, the NU is caused by phenomena such as filtering effects, interference from surrounding sources, and thermal noise [6]. An additional disadvantage of the ED technique is in the lack of ability to distinguish between SU or PU and interference. Regardless of the presented disadvantages, ED is, due to its simple deployment and processing, the most applied SS method in practice [5,7,8]. 

In the ED process, the sensing of the energy of the signal transmitted by the PU in the licensed frequency spectrum is performed. The final goal of this sensing is to determine the test statistics that represent a measure of PU transmission activity. The test statistics are then compared to an in advance set detection threshold (DT). The DT is set from the energy of the noise. The value of the determined DT is the key to performing an accurate ED. The level of DT can be specified as a constant or dynamically adjusted value. The process of DT adjustment enables the dynamic selection of the value of DT according to the NU during the period of SS [3,9,10]. 

To accomplish a better level of detection performance in environments impacted with NU, employing dynamic DT adjustments is a promising solution. However, due to the influence of NU on the signal sensed at the location of SU, the practical realization of DT adjustments in the system exploiting Multiple-Input Multiple-Output (MIMO) Orthogonal Frequency Division Multiplexing (OFDM) transmission is very demanding. In this article, an assessment of the operational efficiency of the ED-based SS in the MIMO-OFDM CR system exploiting the DT adjustment according to NU at the position of SU is presented. The OFDM as technology is widely applied in many communication systems. Recent studies demonstrate that combining the OFDM with MIMO can improve the spectral efficiency in CRNs [11]. The transmission based on MIMO-OFDM technologies has the potential to achieve higher data rates and to alleviate the problem of Inter Symbol Interference in CRNs [12,13,14]. Therefore, combining MIMO and OFDM technologies in CRNs can contribute to the enhancement of spectral efficiency and transmission capacity and investigations dedicated to the performance efficiency of different SS methods in MIMO-OFDM CR systems have been done in [15,16,17]. 

While in Single-Input Single-Output (SISO) transmission systems a single transmission and reception chain or branch (antenna) is used for performing ED, in MIMO systems, multiple transmit (Tx) chains at the PU side and receive (Rx) chains at the SU side are employed. Therefore, the SS employing ED in the MIMO-OFDM CR system can be realized by exploiting various Tx-Rx antenna diversity techniques. Among the different diversity combining techniques, the Square-Law Combining (SLC) technique has the lowest complexity when implemented for the purpose of SS [18]. The ED employing the SLC technique belongs to the non-coherent SS method and its implementation in the MIMO-OFDM CR system does not require channel state information (CSI) for realization of ED.

Hence, ED employing the SLC technique represents a simple and efficient concept for the implementation in the MIMO-OFDM CR system, which motivates presenting the results of the performance analysis of such a concept in this paper. Further motivation is based on the massive exploitation of battery-powered and low-power devices in the emerging Internet of Things (IoT) concept. Massive practical implementation of the IoT concept will be supported with the implementation of the fifth-generation (5G) and upcoming sixth-generation (6G) mobile networks. For enabling the low-power IoT devices to exploit the concept of CR communications, the practical implementation of simple and low complexity SS techniques such as the ED method based on the SLC technique will be of particular interest. The implementation of such an SS technique does not require complex processing or significant device battery depletion, which makes ED based on the SLC technique a promising candidate for massive implementation in future IoT devices equipped with multiple antennas. Hence, the assessment of the ED performance based on the SLC technique in different operating environments affected with NU and performed with DT adjustments is of major significance for possible future realization of such an SS concept in IoT networks. 

Therefore, in this paper, the impact of NU and DT adjustments on the efficiency of the ED employing SLC technique in the SISO and MIMO-OFDM CR systems was analyzed. This paper makes the following contributions:

- The development of the explicit analytic mathematical expressions for the performance assessment of ED process employing SLC method impacted by NU and DT adjustments in MIMO-OFDM CR systems.
- The introduction of the simulation algorithm for executing the ED process by exploiting the SLC method in MIMO-OFDM CR networks affected by different levels of DT adjustments and NUs.
- The comprehensive analyses of simulation results through investigation of the influence of various parameters including the OFDM modulations, the SNRs, the MIMO Tx-Rx chains number, the false alarm probabilities, the Tx powers of PU, the number of sampling points used in ED, and the different levels of NU and DT adjustments on the probability of detection of the PU transmission.

The remaining parts of the paper contain the following sections. A review of the topic associated with the exploitation of the ED method in the MIMO-OFDM CR systems is given in Section 2. Section 3 presents the mathematical expression of the ED principles which involve the effect of DT adjustments and NU on the performance of ED. Section 4 presents a simulation algorithm that enables the ED-based SS in MIMO-OFDM CR systems and the assessment of ED performance impacted with NU and DT adjustments. The comprehensive analysis of the extensive simulation results is given in Section 5. Section 6 concludes the paper. 

## 2. Literature Overview

Table 1 presents the literature survey of related work. In the literature, the performance analysis of CR in MIMO-OFDM systems was performed in [18,19,20,21,22,23]. The authors in [22,23] show that the implementation of the MIMO-OFDM transmission contributes to the enhancement of SS efficiency performed using the ED method. In [22,23,24,25,26], the ED is performed employing the SLC method. In [22], the simulation results indicate that the ED employing SLC can enable precise signal detection for low to moderate SNRs. In [23], SS based on ED and cyclostationary feature detection with and without multiple Tx-Rx chains have been analyzed. The comparative performance results indicate that the Equal Gain Combining (EGC) method requires a precise CSI for performing SS, which consequently results in more efficient SS. On the contrary, the SLC method lacks the need for CSI, which results in lower SS efficiency. In addition, the ED method employing SLC requires detectors and combiners which additionally contribute to the increase in implementation cost. However, the SLC technique is still significantly less complex for implementation when it is compared with other diversity combining schemes which successful operation demands precise knowledge about CSI. 

The challenges related to the hardware implementation of ED employing Square-Law Selection (SLS) and SLC methods are presented in [18]. The authors showed that the proposed solutions can facilitate hardware reliability and antenna diversity in a realistic implementation scenario.

The analyses in [6,27,28] show that the ED performance can be significantly impaired by NU. To improve the ED sensing efficiency degraded by the influence of NU, the authors in [6] proposed a kernelized ED concept based on a DT. For the PU signals affected by Gaussian noise, an assessment of ED performance in communication systems using MIMO transmission have been presented. The results indicate that by increasing the number of sampling points, the number of Rx chains, and SNR at the antennas of SU, the ED method achieves a good level of performance under the Gaussian mixture noise and exceeds the SS efficiency of the classical non-kernelized ED method.

In [29], the subchannel and Tx power allocation concept for downlink communication in multi-cell CR-OFDM access (OFDMA) networks with the adaptive fractional frequency reuse strategy have been proposed. The proposed concept enables maximization of the throughput of the CR SISO network by efficiently assigning OFDM subchannels to base station (BS) cells, while controlling the interference to the PUs through restricting the Tx power on the subchannels used by the PUs. It is shown that the proposed concept improves the system throughput by up to 50% for the same level of interference at locations of PUs. In [30], an even larger improvement of system throughput of 60% and interference reductions have been accomplished. Such improvement is obtained for the proposed licensed shared access (LSA) spectrum sharing framework with the in-band full-duplex transmission in multi-cell multi-user MIMO communication network as the licensee, which operates in the service region of a multi-user MIMO incumbent network. Such significant system throughput improvements have been obtained through the implementation of a hierarchical two-phases beamforming approach, where in the first phase, the LSA controller collects the quality of service demands from the licensee network and based on them, the beamformers are dedicated to reducing the cumulative interference towards the incumbents. In the second phase, the interference minimized in the first step is considered as a constraint for the new beamformer design problem that maximizes the cumulative downlink and uplink data rates of the licensee network. The analyses presented in this paper have been performed for single-cell MIMO-OFDM CR systems exploiting ED based on the SLC technique; however, it can be extended to multi-cell MIMO-OFDM systems.

In [27,28], we analyze the influence of NU on the ED performance in single-input single-output (SISO)-OFDM systems, whose operations are based on rate or margin-adaptive or combined transmission techniques. The obtained results indicate that the ED will predominantly be impacted by NU. To reduce the effect of NU, in [28] the analysis of ED performance exploiting DT adjustments in the SISO communications systems is performed. The obtained results indicate that the ED method can be improved by involving dynamic adjustments of the DT in the ED impacted with NU. Furthermore, in [31], a simulating algorithm that enabled the performance analysis of the ED method employing SLC in MIMO-OFDM CR systems is proposed. Based on the algorithm proposed, in [32] we present the preliminary results of the performance assessment of the ED method employing SLC in the MIMO communications systems. The results show that increasing the number of Rx and Tx chains in the MIMO system give a contribution to the enhancement of ED performance. 

Although the earlier research works [18,22,23,24,25,26,31,32] show that using the ED method employing the SLC technique provides a positive contribution to the improvement of the efficiency of the ED, an investigation into how NU and DT adjustments impact the ED sensing efficiency in MIMO-OFDM CRs systems is missing. Therefore, in this paper for the first time, the mathematical equations which express how the NU and dynamic DT adjustments, the number of PU Tx and SU Rx chains, the probability of false alarms, the SNRs, and the number of sampling points impact the ED performance in the MIMO-OFDM CR system have been developed. In addition, an extensive analysis of the impact of NU and DT adjustments on the ED employing the SLC technique in MIMO-OFDM CR systems is presented in this paper. The presented analyses establish the basis for understanding the ED operation exploiting the capability of the dynamic DT adjustments in the MIMO-OFDM CR systems affected by NU.

## 3. System Design and Explanation of the Energy Detection Operation 

**Figure 1:**
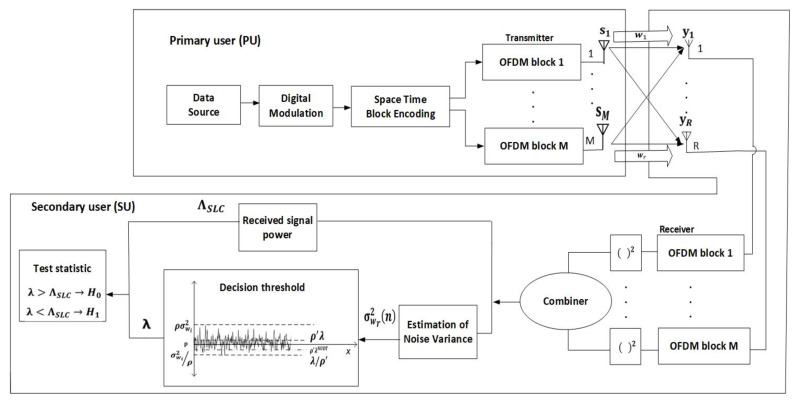
Main blocks of the MIMO-OFDM wireless communication system for SS based on ED employing SLC technique.

The visualization of the block schema of the analyzed single-cell MIMO-OFDM CR system consisted of single PU and single SU is presented in Figure 1. The signal in the analyzed MIMO-OFDM CR system is transmitted using space-time block codes (STBCs) [33]. The PU is a licensed user and it has a higher priority when using the dedicated frequency spectrum. The SU has a lower priority and opportunistically accesses the spectrum in the absence of PU [3]. The SU is permitted to use the spectrum in a way that does not cause interference to the PUs. For that reason, the SU performs SS using the ED method employing the SLC technique (Figure 1). In the process of SLC, the SU equipped with an energy detector performs a signal squaring operation in the square-law device which is followed by combining the squared signals received at *R* Rx chains in the finite time integrator (Figure 1). 

The PU Tx power in the MIMO system emitted over the *m*-th Tx chain (antenna) is formulated as *P_m_*. The $P=\sum_{m=1}^{M}P_{m}$ defines the overall instant Tx power of the PU transmitted via *M* Tx chains (Figure 1). Table 2 lists the descriptions of all parameters used in the analysis. The complex signal defined as $s_{m}=s_{m,r}+js_{m,i}$ is assumed as the signal transmitted via *m*-th Tx chain of PU (Figure 1). Hence, the signals carried via the *M* Tx chains of PU are expressed as $s=\sum_{m=1}^{M}s_{m}$. The signal received by the SU at every *R* Rx chain (antenna) and sampled by *n* samples where *n* = 1,…, *N* can be formulated as:

(1) $$y_{r}\left(n\right)=\{\begin{array}{c} w_{r}\left(n\right)\\ h_{r\;}\left(n\right)\;s_{r}\left(n\right)+w_{r}\left(n\right) \end{array}$$

The $h_{r\;}\left(n\right)$ is of size ${\mathbb{C}}^{1XM}$ and it is a complex vector that represents the wireless channel gain among the *M* Tx chains of PU and *r*-th Rx chain of SU (Figure 1). Complex vector $s_{r}\left(n\right)$ is of size ${\mathbb{C}}^{MX1}$ and represents the Tx signal of PU that is received at *r*-th Rx chain of the SU in *n*-th sample (sensing moment).

Additionally, the complex noise signal $w_{r}\left(n\right)$ is the additive white Gaussian noise (AWGN) impacting the signal received at the *r*-th Rx chain of the SU. The impact of noise is assumed to be an identically distributed and independent random process having a mean value equal to zero and a variance of $\sigma_{w}^{2}$, which distribution is circularly symmetric and defined as $N\;\left(0,\;2\sigma_{w}^{2}{}_{r}\left(n\right)\right)$.

Since the PU signal is impacted by noise, the SNR at the SU *r*-th chain is formulated as:

(2) $$SNR_{r}\left(n\right)=\gamma_{r}\left(n\right)=\frac{{\left|h_{r}\left(n\right)\right|}^{2}\;\frac{1}{N}\;\sum_{n=1}^{N}{\left|\;s_{r}\left(n\right)\right|}^{2}}{2\sigma_{w}^{2}{}_{r}\left(n\right)}$$

The total *SNR* at all *M* Rx chains (antennas) during the *n*-th SS period is expressed as: $\gamma_{SLC}=\sum_{r=1}^{R}\gamma_{r}\left(n\right)$. Furthermore, the mean SNR value at the antenna(s) of SU for all *R* Rx chains in the *n*-th sampling period is defined as: $\bar{\gamma_{SLC}}=\frac{1}{R}\sum_{r=1}^{R}\gamma_{r}\left(n\right)\;$=$\;\frac{1}{R}\gamma_{SLC}$. 

Spectrum sensing is the process where the SUs continuously supervise the activity of the PUs in order to detect the spectrum holes. Detailed knowledge about spectrum availability obtained through the testing of a binary hypothesis $H_{0}$ and $H_{1}$ shown in Equation (3), represents the fundamental operation of the ED. This process aims to decide between two hypotheses, according to which the PU signal is assumed to be absent (denoted as $H_{0}$) or the PU signal is assumed to be present (denoted by $H_{1}$). Detailed knowledge about spectrum availability obtained through the testing of a binary hypothesis $H_{0}$ and $H_{1}$ shown in Equation (3), represents the fundamental operation of the ED. Therefore, the decision on SS occupancy is the result of the testing of the following hypothesis:

(3) $$Y\left(n\right)=\{\begin{array}{c} \overset{R}{\underset{r=1}{\sum }}w_{r}\left(n\right):\;\;H_{0}\;\\ \overset{R}{\underset{r=1}{\sum }}h_{r\;}\left(n\right)\;s_{r}\left(n\right)+\overset{R}{\underset{r=1}{\sum }}w_{r}\left(n\right):\;\;\;H_{1} \end{array}$$

where Y(n) is the total signal detected from all *R* Rx chains in the *n*-th SS period at a position of SU. The focus of the ED is making a decision on whether the detected signal ***Y***(*n*) satisfies hypotheses $H_{0}$ or $H_{1}$. Therefore, in the process of deciding whether the PU is present or not, the threshold is compared with the sensed energy of the signal detected at the antennas of SU. The decision hypotheses $H_{1}$ is satisfied when the sensed energy of the signal detected at the antennas of SU is greater than the threshold. This results in the conclusion that the PU transmits in the dedicated band. The decision hypotheses $H_{0}$ is satisfied if the energy of the detected signal is lower than the DT. This results in the cognition that the signal of PU is absent. Thus, the result of this binary hypothesis test determines the SU activity in the terms of possible transmission in the PU frequency band. 

### 3.1. Process of Energy Detection

The procedure of ED employing SLC technique in MIMO-OFDM CR systems aims to exploit the SS of the PU signal by means of all *R* Rx chains of SU. In accordance with the SLC method, the signals received on all *R* Rx chains are squared and combined to get the total received signal energy known as the test statistic. The overall test statistic can therefore be expressed as:

(4) $$\Lambda_{SLC}=\overset{R}{\underset{r=1}{\sum }}\Lambda_{r}=\overset{R}{\underset{r=1}{\sum }}\overset{N}{\underset{n=1}{\sum }}{\left|y_{r}\left(n\right)\right|}^{2}\;$$

where the test statistics of the SU *r*-th Rx chain is expressed as $\Lambda_{r}$.

The decision regarding spectrum occupancy by the PU is performed through the comparison of the test statistic with a DT (λ(n)) that is dynamically selected for each sample *n* used in the SS process:

(5) $${\;\Lambda }_{SLC}\left(n\right)>\lambda \left(n\right):\;H_{0},\;\forall n\in \left\{1,\;\ldots ,\;N\right\}\;$$

(6) $$\Lambda_{SLC}\left(n\right)>\lambda \left(n\right):\;H_{1},\;\forall n\in \left\{1,\;\ldots ,\;N\right\}$$

In accordance with [26,34], for an adequate number of sampling points *N*, the distribution of the total test statistic given in Equation (4) can be approximated using a normal distribution as:

(7) $$\Lambda_{SLC}\sim N\left(\begin{array}{c} \sum_{r=1}^{R}\sum_{n=1}^{N}E\left[{\left|y_{r}\left(n\right)\right|}^{2}\right],\;\;\;\\ \sum_{r=1}^{R}\sum_{n=1}^{N}Var\left[{\left|y_{r}\left(n\right)\right|}^{2}\;\right] \end{array}\right),$$

where E [·] expresses the expectation operator and Var [ · ] expresses the variance operator. 

In each observed SS period *n*, the uniform gain of the wireless channel $h_{r}\left(n\right)$ and the noise variance of the detected signal at the *r*-th Rx chain (antenna) $2\sigma_{w}^{2}{}_{r}\left(n\right)$ can be formulated as:

(8) $$h_{r}\left(n\right)=h\;\;\forall r=1,\;\ldots ,\;R;\;\forall n=1,\;\ldots ,\;N$$

(9) $$2\sigma_{w}^{2}{}_{r}\left(n\right)=2\sigma_{w}^{2}\;\forall r=1,\;\ldots ,\;R;\;\forall n=1,\;\ldots ,\;N$$

where h represents the complex matrix of the channel gain for all *R* Rx chains.

Performing the SS using the ED method employing SLC demands knowledge about the average level of the power received at the antennas of SU Rx chains. Therefore, the overall instant PU Tx power of the signal transmitted using *M* Tx chains (antennas) within the *n*-th observation period, is equal to all signal variances at the *r*-th Rx chain of the SU. It can be formulated as$\;\;P=\sum_{r=1}^{R}{\left|h\right|}^{2}2\sigma_{s_{r}}^{2}\left(n\right)$. The interdependence among the mean values of SNR at the antennas of SU and the instant Tx power of the PU can be approximated with $\bar{\gamma_{SLC}}\approx \frac{P}{R2\sigma_{w}^{2}}$. 

Considering the above assumptions, the total test statistics from Equation (7) can be approximated with:

(10) $$\Lambda_{SLC}\sim \{\begin{array}{r} N\;\left(RN\left(2\sigma_{w}^{2}\right),\;RN{\left(2\sigma_{w}^{2}\right)}^{2}\right):\;\;H_{0}\;\\ N\left(N\left(2\sigma_{w}^{2}\right)\left(R+\gamma_{SLC}\right),\;N{\left(2\sigma_{w}^{2}\right)}^{2}\left(R+2\gamma_{SLC}\right)\right):\;\;\;H_{1} \end{array}$$

According to the overall test statistic presented in Equation (10) for hypotheses *H*_1_ and *H*_0_, the false alarm and detection probability for ED SS employing SLC diversity technique in MIMO-OFDM CR systems was developed. 

### 3.2. Probabilities of False Alarm and Detection for MIMO-OFDM CR Systems 

The performance of ED SS techniques is exploited through two probabilities: detection probability ($P_{di}$) and false alarm probability ($P_{fa})$. The probability of sensing the transmitted PU signal at the position of SU when it is really transmitted is known as the probability of detection ($P_{d}$). It can be examined through the verification of hypothesis $H_{1}$ as $P_{d}[\mathrm{Pr}(\Lambda_{SLC}>\lambda )\;|H_{1}]$. The detection probability for the ED method employing SLC technique in MIMO communication systems can be defined as

(11) $$\begin{array}{ccc} P_{d}[\mathrm{Pr}(\Lambda_{SLC}>\lambda )\;|H_{1}] & \approx & Q\left(\frac{\lambda_{d}-N\left(2\sigma_{w}^{2}\right)\left(R+\gamma_{SLC}\;\right)}{\sqrt{N\left(R+2\gamma_{SLC}\right)}\;\left(2\sigma_{w}^{2}\right)}\right)\approx Q\left(\frac{\lambda_{d}-RN\left(2\sigma_{w}^{2}\right)\left(1+\bar{\gamma_{SLC}}\;\right)}{\sqrt{RN\left(1+2\bar{\gamma_{SLC}}\right)}\;\left(2\sigma_{w}^{2}\right)}\right)\\ & \approx & Q\left(\frac{\lambda_{d}-RN\left(2\sigma_{w_{i}}^{2}\right)\left(1+\frac{P}{2R\sigma_{w}^{2}}\;\right)}{\sqrt{RN\left(1+\frac{P}{{R\sigma }_{w}^{2}}\right)}\;\left(2\sigma_{w}^{2}\right)}\right) \end{array}$$

where Q (.) is the Gaussian-Q function and $\lambda_{d}$ represents the DT level. For better PU signal detection, a higher detection probability ($P_{d})$ is required. A higher detection probability ($P_{d}$) improves the usage of spectrum and ED performance of the SU.

The false alarm probability ($P_{fa}$) is the probability of sensing a PU signal by the SU, when the signal of PU is not really transmitted. It can be examined through the verification of hypothesis *H*_0_ as $P_{fa}[\mathrm{Pr}(\Lambda_{SLC}>\lambda \;)\;|H_{0}]$. The false alarm probability ($P_{fa}$) as a performance metric of ED employing the SLC method in MIMO communication systems can be defined as:

(12) $$P_{fa}[\mathrm{Pr}(\Lambda_{SLC}>\lambda \;)\;|H_{0}]\approx Q\left(\frac{\lambda_{fa}-RN\left(2\sigma_{w}^{2}\right)}{\sqrt{RN}\;\left(2\sigma_{w}^{2}\right)}\right)$$

where $\lambda_{fa}$ represents the false alarm threshold level. According to Equations (11) and (12), the detection probability and false alarm probability depend on the number of sampling points (*N*), the variance of noise ($\sigma_{w}^{2}$), the number of Rx chains (*R*) of SU, and the level of the defined detection or false alarm thresholds. Additionally, Equation (11) indicates that the detection probability will also depend on the level of PU Tx power *P*.

From the perspective of the SU, a lower $P_{fa}$ means more chances that the channel can be reused when it is available. This consequently results in the possibility of achieving higher throughputs for the SU. Thus, a fundamental trade-off between the sensing capability and achievable throughput of the SU exists for SS based on ED. Therefore, a reliable energy detector should ensure a low false alarm probability ($P_{fa})\;$and a high detection probability ($P_{d}$). This means that appropriate QoS for a SU when using a wireless network should be provided, while also establishing an appropriate level of PU protection during periods of transmitting should be guaranteed. 

From Equations (11) and (12), it is possible to define the minimum number of sampling points ($N_{min}$) needed for achieving the precise detection of the PU signal, which is expressed as:

(13) $$N_{min}=\frac{{\left[\sqrt{R}Q^{-1}\left(P_{fa}\right)-\sqrt{\left(R+2\gamma_{SLC}\right)}Q^{-1}\left(P_{d}\right)\right]}^{2}}{\gamma_{SLC}{}^{2}}=\frac{{\left[Q^{-1}\left(P_{fa}\right)-\sqrt{\left(1+2\bar{\gamma_{SLC}}\right)}Q^{-1}\left(P_{d}\right)\right]}^{2}}{R{\bar{\gamma_{SLC}}}^{2}}$$

Equation (13) indicates that the calculation of the minimum number of sampling points does not request knowledge of the DT level. For the number of sampling points higher than the minimal, the detection of the PU signal will be ensured for any DT level. 

Furthermore, it is already known that for low SNR at the antennas of SU, a high number of sampling points is required for precise sensing of PU signal. The need for a larger number of sampling points results in a larger sensing duration, which negatively affects the battery discharge of battery-powered devices. By combining (11) and (12) and considering that in practice $\lambda_{d}$ = $\lambda_{fa}$ = λ, the correlation between a detection and false alarm probability can be formulated as

(14) $$P_{d}=Q\left(\frac{Q^{-1}\left(P_{fa}\right)-\sqrt{\frac{N}{R}}\gamma_{SLC}}{\sqrt{\left(1+2\frac{\gamma_{SLC}}{R}\;\right)}\;}\right)=Q\left(\frac{Q^{-1}\left(P_{fa}\right)-\sqrt{RN}\;\bar{\gamma_{SLC}}}{\sqrt{\left(1+2\;\bar{\gamma_{SLC}}\right)}\;}\right)\;=Q\left(\frac{Q^{-1}\left(P_{fa}\right)-\frac{\sqrt{N}P}{2\sqrt{R}\sigma_{w}^{2}}}{\sqrt{\left(1+\frac{P}{R\sigma_{w}^{2}}\right)}\;}\right)$$

Based on Equation (14), it can be seen that the detection probability can be expressed without a DT level, if the targeted probability of false alarm is known. An approach based on defining the operation of the CR network by setting a constant false alarm probability is known as the constant false alarm rate (CFAR) ED approach. 

### 3.3. Detection Threshold Estimation 

According to what has been presented in the previous section, the false alarm and detection probabilities are affected by the corresponding threshold values. Determining the level of DT is the main activity that influences the decision efficiency regarding the absence or presence of the PU signal. The fixed detection threshold (FDT) and the dynamic detection threshold (DDT) methods are mainly considered in the literature as two opposing approaches for the selection of DTs in the ED process. In the case of FDT, the set threshold has a constant value even when the signal fluctuations in the frequency channel are present. However, to take into account fluctuations in the state of the wireless channel, the method based on DDT adjustments has been introduced. In the DDT method, the level of DT is adjusted to the channel conditions to maximize the detection probability. The literature indicates that the DDT method provides better SS performance compared to that of the FDT method [35]. In addition, a well-chosen level of DT can minimize the SS error, ensuring that there is enough protection in the usage of the licensed band of the PU, which further contributes to enhancing the spectrum utilization [36].

According to (12), for a given noise variance $\sigma_{w}^{2}$, the number of Rx chains and the number of sampling points *N* used for SS in the ED process, the FDT level can be calculated for the fixed false alarm probability according to the following:

(15) $$\lambda_{fa}=\left[Q^{-1}\left(P_{fa}\right)+\sqrt{RN}\right]\sqrt{RN}2\sigma_{w}^{2}$$

Hence, such an approach known as the CFAR approach calculates the value of the false alarm threshold that needs to maximize the probability of detection. This CFAR approach is used for SS employing ED in systems that require the maximal utilization of the wireless channel.

Alternatively, to provide an appropriate priority and sufficient protection of the transmissions performed by PU, the selection of the DT based on the constant detection rate (CDR) can be defined from Equation (11):

(16) $$\lambda_{d}=\left[Q^{-1}\left(P_{d}\right)\sqrt{\left(1+\frac{P}{R\sigma_{w}^{2}}\right)}+\sqrt{RN}\;\left(1+\frac{P}{2{R\sigma }_{w}^{2}}\right)\right]\sqrt{RN}2\sigma_{w}^{2}$$

The CDR approach in SS based on ED is applied when it is important to eliminate the interference in the observed CR system. 

By comparing Equations (15) and (16), it can be noticed that the CFAR approach does not request the information about the total instant PU Tx power (*P*) of the signal transmitted over *M* Tx chains. This makes the CFAR approach more applicable in practical implementations. Although the CFAR compared to the CDR approach improves the throughput of the SU in the CR systems, the CFAR approach is unable to ensure the adequate protection of the PU transmission in comparison to the CDR approach.

In addition, selecting a low value of false alarm probability ($P_{fa}$) results in need for a high level of the corresponding threshold ($\lambda_{fa})$. As a consequence, an interference among PU and SU can appear and the setting of the fixed false alarm threshold $\lambda_{fa}$ based on the CFAR approach is not the most favorable. A more favorable approach is seen in the DDT adjustments based on dynamic changes in the level of DT according to the wireless channel conditions. 

However, the DDT adjustments according to the conditions in the wireless channel between PU and SU are highly challenging when it comes to practical realization. In this work, to mathematically model the DDT adjustments, the DDT factor $\rho^{\prime }$ ($\rho^{\prime }\geq 1$) is used. The DDT factor $\rho^{\prime }$ defines the level of DDT adjustments. The higher values of the DDT factor enable the modeling of the higher adjustment capabilities of the ED according to changes in the state of the wireless channel. Hence, instead of being permanently fixed as in the case of ED with FT, the dynamically selected DT values may be in the interval [λdDTρ′, ρ′λdDT].

When the ED employing SLC is performed with a DDT adaptation, the probability of detection ($P_{d}^{DT}$) and false alarm probability ($P_{fa}^{DT}$) is expressed as:

(17) PdDT=minλ′DTϵ[λdDTρ′, ρ′λdDT]Q(λ′DT−RNDT(2σw2)(1+P2Rσw2 )RNDT(1+PRσw2) (2σw2))=Q(λdDTρ′ −RNDT(2σw2)(1+P 2Rσw2 )RNDT(1+PRσw2) (2σw2))

(18) PfaDT=maxλ′DTϵ[λfaDTρ′   ρ′λfaDT]Q(λ′DT−RNDT(2σw2)RNDT (2σw2))=Q(ρ′λfaDT−RNDT(2σw2)RNDT (2σw2))

where $N^{DT}$ and $\lambda_{d}^{DT}$ represents the number of sampling points and the level of DDT used in the ED exploiting DDT adjustments, respectively. Based on Equation (17), the level of DDT can be derived and expressed as:

(19) $$\lambda_{d}^{DT}=\left[Q^{-1}\left(P_{d}^{DT}\right)\sqrt{\left(1+\frac{P}{R\sigma_{w}^{2}}\right)}+\sqrt{RN}\left(1+\frac{P}{2R\sigma_{w}^{2}}\right)\right]\sqrt{RN^{DT}}\left(2\sigma_{w}^{2}\right)\rho^{\prime }$$

Similarly, the level of false alarm threshold can be derived from (18) and formulated as:

(20) $$\lambda_{fa}^{DT}=\left[Q^{-1}\left(P_{fa}^{DT}\right)+\sqrt{RN^{DT}}\right]\sqrt{RN^{DT}}\left(\frac{2\sigma_{w}^{2}}{\rho^{\prime }}\right)$$

A minimal number of sampling points ($N^{DT})\;$for performing exact ED employing the SLC technique in MIMO systems with an implemented DDT, can be realized by adapting Equation (13) as follows:

(21) $$N^{DT}=\frac{{\left[Q^{-1}\left(P_{fa}^{DT}\right)-\sqrt{\rho^{\prime 2}\left(1+2\bar{\gamma_{SLC}}\right)}Q^{-1}\left(P_{d}^{DT}\right)\right]}^{2}}{R{\left[\rho^{\prime 2}\bar{\gamma_{SLC}}+(\rho^{\prime 2}-1)\right]}^{2}}$$

The correlation between the probability of false alarm and detection in the case of systems performing ED with DDT adjustments can be formulated by combining Equations (17) and (18), and it can be expressed as:

(22) $$P_{d}^{DT}=Q\left(\frac{Q^{-1}\left(P_{fa}^{DT}\right)-\sqrt{RN^{DT}}\left(\rho^{\prime 2}\bar{\gamma_{SLC}\;}+\left(\rho^{\prime 2}-1\right)\right)}{\sqrt{\rho^{\prime 2}\left(1+2\bar{\gamma_{SLC}}\right)}\;}\right)\;=Q\left(\frac{Q^{-1}\left(P_{fa}^{DT}\right)-\sqrt{RN^{DT}}\left(\rho^{\prime 2}\frac{P}{2R\sigma_{w}^{2}}+\left(\rho^{\prime 2}-1\right)\right)}{\sqrt{\rho^{\prime 2}\left(1+\frac{P}{R\sigma_{w}^{2}}\right)}\;}\right)\;=Q\left(\frac{Q^{-1}\left(P_{fa}^{DT}\right)-\frac{\rho^{\prime 2}P\sqrt{N^{DT}}}{2\sqrt{R}\sigma_{w}^{2}}-\sqrt{RN^{DT}}\left(\rho^{\prime 2}-1\right)\;}{\sqrt{\rho^{\prime 2}\left(1+\frac{P}{R\sigma_{w}^{2}}\right)}\;}\right)$$

For the case where the DDT factor $\rho^{\prime }\;$= 1, Equations (17), (18) and (22) are equal to Equations (11), (12) and (14), respectively. In the case where the DDT factor $\rho^{\prime }$ > 1, the process of signal detection is based on the DDT adjustments. For the larger values of the DDT factor, the selection of a larger range of DDT levels is possible. 

### 3.4. Estimation of Noise Uncertainty 

As shown in Equations (15) and (16), the calculation of false alarm and detection thresholds depends on the levels of noise variance $\sigma_{w}^{2}$. However, ED in a wireless MIMO-OFDM-based communication system is performed by exploiting an estimation of noise power. The lack of knowledge about noise fluctuations significantly contributes to the limited knowledge about the properties of the AWGN. This phenomenon of unknown random variations in the noise power $\sigma_{w}^{2}$ is known as NU. The NU negatively affects the ED performance in terms of reducing the precision of SS accuracy. 

To have more realistic conditions for the performance analysis of ED sensing efficiency in the MIMO wireless communication system, the impact of the NU variations on the PU signal detection was included in the analysis presented in this paper. The impact of the fluctuations in noise power on ED performance is characterized by the NU factor (ρ). The NU factor ρ (ρ≥1) is a positive parameter that defines the scope of the NU. To express the influence of NU on the sensing efficiency of the ED, the boundaries of the noise variance ($\sigma_{wNU}^{2}$) are defined by the finite interval σwNU2ϵ[σnw2ρ, ρσw2]. Based on Equations (11) and (12), the detection ($P_{d}^{NU}$) and false alarm ($P_{fa}^{NU}$) probabilities of the PU signal impacted by NU can be expressed for the ED method employing the SLC technique as:

(23) PdNU(Pr(ΛSLC<λ) |H1)=minσwNU2ϵ[σw2ρ, ρσw2]Q(λ − RNNU(2σwNU2)(1 + P2RσwNU2 )RNNU(1 + PRσwNU2) (2σwNU2))=Q(λdNU − RNNU(2σw2ρ)(1 + Pρ2Rσw2 )RNNU(1 + PρRσw2) (2σw2ρ))

(24) PfaNU(Pr(ΛSLC>λ)|H0)=maxσwNU2ϵ[σw2ρ,ρσw2]Q(λ−RNNU(2σwNU2)RNNU (2σwNU2))=Q(λfaNU−RNNU(2ρσw2)RNNU (2ρσw2))

where $\lambda_{d}^{NU}$ represents the DT, $\lambda_{fa}^{NU}$ represents the false alarm threshold and $N^{NU}$ represents the number of sampling points used in the ED process impacted with NU. Equations (23) and (24) indicate that the total number of receiving chains (*R*) of SU, the number of sampling points ($N^{NU}$) and the NU variance ($\sigma_{wNU}^{2}$) with NU factor ρ are the parameters that influence both, the detection and false alarm probability in MIMO communication systems. Besides these parameters, the total instant Tx power of the PU signal (*P*) impacts the detection probability. Based on Equations (23) and (24), it can be noticed that setting appropriate DT or false alarm thresholds will significantly impact the detection and false alarm probabilities. 

Based on Equations (23) and (24), the DT and false alarm threshold can be expressed as:

(25) $$\lambda_{d}^{NU}=\left[Q^{-1}(P_{d}^{NU})\sqrt{\left(1+\frac{P\rho }{R\sigma_{w}^{2}}\right)}+\sqrt{RN^{NU}}\left(1+\frac{P\rho }{2R\sigma_{w}^{2}}\right)\right]\sqrt{RN^{NU}}\left(\frac{2\sigma_{w}^{2}}{\rho }\right)$$

(26) $$\lambda_{fa}^{NU}=\left[Q^{-1}(P_{fa}^{NU})+\sqrt{RN^{NU}}\;\right]\sqrt{RN^{NU}}\;\left(2\rho \sigma_{w}^{2}\right)$$

According to Equation (13), the minimal number of sampling points $N^{NU}$ used in the ED which guarantees the accurate detection of the PU signal affected by NU can be formulated as

(27) $$N^{NU}=\frac{{\left[\rho Q^{-1}\left(P_{fa}^{NU}\right)-\sqrt{\left(\frac{1}{\rho }+2\bar{\gamma_{SLC}}\right)}Q^{-1}\left(P_{d}^{NU}\right)\right]}^{2}}{R{\left(\bar{\gamma_{SLC}}-\frac{\rho -1}{\rho }\right)}^{2}}$$

Equation (27) indicates that if the average SNR at antennas of SU is lower than the $\frac{\rho^{2}-1}{\rho }$, the energy detector cannot sense the signal. For that reason, the SNR level at the antennas of SU will have an important role in the accurate detection of PU signals. 

According to Equations (22) and (23), when the ED is impacted by NU, the detection probability can be defined as a function of the false alarm probability:

(28) $$P_{d}^{NU}=Q\left(\frac{\rho Q^{-1}\left(P_{fa}^{NU}\right)-\sqrt{RN^{NU}}\left(\bar{\gamma_{SLC}}-\frac{\rho -1}{\rho }\right)}{\sqrt{\frac{1}{\rho }+2\bar{\gamma_{SLC}}}\;}\right)=Q\left(\frac{\rho Q^{-1}\left(P_{fa}^{NU}\right)-\sqrt{RN^{NU}}\left(\frac{P}{2R\sigma_{w}^{2}\;}-\frac{\rho -1}{\rho }\right)}{\sqrt{\frac{1}{\rho }+\frac{P}{R\sigma_{w}^{2}}}\;}\right)\;=Q\left(\frac{\rho Q^{-1}\left(P_{fa}^{NU}\right)-\frac{\sqrt{N^{NU}}P}{2\sqrt{R}\sigma_{w}^{2}\;}+\sqrt{RN^{NU}}\left(\frac{\rho -1}{\rho }\right)\;}{\sqrt{\frac{1}{\rho }+\frac{P}{R\sigma_{w}^{2}}}\;}\right)$$

The value of NU factor ρ = 1.00 indicates that fluctuations in noise power do not exist and that there is no impact of the NU on the ED process. In this case, Equations (22), (23) and (28) converge into (11), (12) and (14). The modelling of the influence of the NU on the ED is performed for the case where the NU factor ρ > 1.00. A higher value (than one) of the NU factor ρ means larger NU fluctuations, for which it is expected to have a more negative impact on the performance of the ED SS.

### 3.5. Energy Detection Process with NU and DT 

An approach that can contribute to the minimization of the negative effects caused by NU on the efficiency of the ED is dedicated to involving the DT adjustments during the ED. This can contribute to the enhancement of SS performance of the ED method. The ED employing SLC which encompasses the DT adjustments for reducing the impact of NU, represents the most realistic approach to the analysis of the performance of ED in the MIMO-OFDM CR systems. However, this approach is the most difficult for either simulations or practical implementation due to the necessity of the continuous estimation of NU and the dynamic adjustments of DT during the ED according to the NU.

For the analysis of this ED approach, detection and false alarm probabilities are expressed as a function of NU variance and the DT adjustment factors. The boundaries of the NU are assumed to be in the interval σwNUDT2ϵ[σw2ρ, ρσw2], while the boundaries of the DTs are assumed to be in the interval λ′NUDTϵ[λdNUDTρ′, ρ′λdNUDT]. Considering these boundaries, the probabilities of false alarm ($P_{fa}^{NUDT}$) and detection ($P_{d}^{NUDT})$ for the ED approach which includes the impact of NU and DT adjustments can be formulated as:

(29) PdNUDT=minλ′NUDTϵ[λdNUDTρ′ ρ′λdNUDT]  minσwNUDT2ϵ[σw2ρ, ρσw2] Q(λi′NUDT−RNNUDT(2σwNUDT2)(1+P2RσwNUDT2 )RNNUDT(1+PRσwNUDT2) (2σwNUDT2))=Q(λdNUDTρ′−RNNUDT(2σw2ρ)(1+Pρ2Rσw2 )RNNUDT(1+PρRσw2) (2σw2ρ))

(30) PfaNUDT=maxλ′NUDTϵ[λfaiNUDTρ′,ρ′λfaNUDT]                   maxσwNUDT2ϵ[σwi2ρ,ρσw2]Q(λ′NUDT−RNNUDT(2σw2)RNNUDT (2σw2))=Q(λfaNUDTρ′−RNNUDT(2σw2)ρRNNUDT (2σw2)ρ)

The level of DT ($\lambda_{d}^{NUDT}$) can be derived from (29) as:

(31) $$\lambda_{d}^{NUDT}=\left[Q^{-1}\left(P_{d}^{NUDT}\right)\sqrt{\left(1+\frac{P\rho }{R\sigma_{w}^{2}}\right)}+\sqrt{RN^{NUDT}}\left(1+\frac{P\rho }{2R\sigma_{w}^{2}}\right)\right]\sqrt{RN^{NUDT}}\;\left(\frac{2\sigma_{w}^{2}\rho^{\prime }}{\rho }\right)$$

Similarly, for the defined value of false alarm probability, the corresponding threshold can be derived from (30) as:

(32) $$\lambda_{fa}^{NUDT}=\left[\;Q^{-1}\left(P_{fa}^{NUDT}\right)+\sqrt{RN^{NUDT}}\right]\;\frac{2\rho \sigma_{w}^{2}}{\rho^{\prime }}\sqrt{RN^{NUDT}}$$

In addition, the minimal number of sampling points for the successful detection of PU signal when ED is impacted by NU and performed based on DT adjustments can be formulated as:

(33) $$N^{NUDT}=\frac{{\left[\frac{\rho }{\rho^{\prime }}Q^{-1}\left(P_{fa}^{NUDT}\right)-\sqrt{\rho^{\prime }\left(\frac{1}{\rho }+2\bar{\gamma_{SLC}}\right)}Q^{-1}\left(P_{di}^{NUDT}\right)\right]}^{2}}{R{\left[\rho^{\prime }\bar{\gamma_{SLC}}+\frac{\rho^{\prime }}{\rho }-\frac{\rho }{\rho^{\prime }}\right]}^{2}}$$

By combining Equations (28) and (29), the detection probability can be expressed as a function of false alarm probability as

(34) $$P_{d}^{NUDT}=Q\left(\frac{\frac{\rho }{\rho^{\prime }}Q^{-1}\left(P_{fa}^{NUDT}\right)-\sqrt{RN^{NUDT}}\left(\rho^{\prime }\;\bar{\;\gamma_{SLC}}+\frac{\rho^{\prime }}{\rho }-\frac{\rho }{\rho^{\prime }}\right)}{\sqrt{\rho^{\prime }\left(\frac{1}{\rho }+2\bar{\gamma_{SLC}}\right)}\;}\right)\;=Q\left(\frac{\frac{\rho }{\rho^{\prime }}Q^{-1}\left(P_{fa}^{NUDT}\right)-\sqrt{RN^{NUDT}}\left(\rho^{\prime }\;\frac{P}{2R\sigma_{w}^{2}}+\frac{\rho^{\prime }}{\rho }-\frac{\rho }{\rho^{\prime }}\right)}{\sqrt{\rho^{\prime }\left(\frac{1}{\rho }+\frac{P}{R\sigma_{w}^{2}}\right)}\;}\right)\;=Q\left(\frac{\frac{\rho }{\rho^{\prime }}Q^{-1}\left(P_{fa}^{NUDT}\right)-\frac{\rho^{\prime }\sqrt{N^{NUDT}}P}{2\sqrt{R}\sigma_{w}^{2}}-\sqrt{RN^{NUDT}}\left(\frac{\rho^{\prime }}{\rho }-\frac{\rho }{\rho^{\prime }}\right)}{\sqrt{\rho^{\prime }\left(\frac{1}{\rho }+\frac{P}{R\sigma_{w}^{2}}\right)}\;}\right)$$

Equation (34) indicates that the NU and DDT factors have an affect on the detection probability when SS is performed in MIMO-OFDM cognitive radio systems based on ED. The joint impact of NU and DT adjustments on sensing performance can be modeled by selecting the corresponding values of DDT and NU factors ($\rho^{\prime }$ > 1.00 and ρ > 1.00). In the case of the DDT and NU factors equal to $\rho^{\prime }$ = 1.00 and ρ > 1.00, the channel is impacted by NU and there are no DDT adjustments during the ED process. For the same conditions in the channel and the same number of sampling points ($N^{NU}=N^{NUDT}$) used during the ED impacted by NU, Equations (29), (30) and (34) converge into Equations (23), (24) and (28), respectively. In case of $\rho^{\prime }$ > 1.00 and ρ = 1.00, the ED includes DT adjustments during the SS without an impact from NU. For the same conditions in the channel and the same number of sampling points ($N^{DT}=N^{NUDT}$) used in ED performed with DT adjustments, Equations (29), (30) and (34) converge into Equations (17), (18) and (22), respectively. 

## 4. Simulation Algorithm for the ED Employing SLC

The ED performance employing the SLC technique in the MIMO-OFDM CRs systems has been tested through the executing the proposed simulation Algorithm 1. The algorithm is composed of two phases. In the first phase, the procedure of ED is executed according to the principles of the concept employing SLC of the signals received at the *R* RX chains of SU. In the second phase, to simulate the behavior of ED performance in different operating environments, the execution of the algorithm was continued through the selection of the impact of different combinations of NU and DDT factors on ED performance. Table 3 indicate parameters used in the process of simulation.
**Algorithm 1** Simulation of the ED in distinct working environments of MIMO-OFDM CR systems. *1: **INPUT:** MIMO_OFDM_M×r, noise variance* ($\sigma_{w}^{2})$*, number of sampling points* (*N*)*, probability of false alarm* ($P_{fa}$)*, number of Monte Carlo simulations*(*pp*)*,*
*SNR simulation range* (*SNR*)*, length of the MIMO-OFDM data* (*mimo_ofdm_len*)*, DDT factor* (ρ′)*, and NU factor* (*ρ*)*,* *2: **OUTPUT:** Detection probability impacted by DT adjustment* ($P_{d}^{DT}$) *and Detection probability impacted by NU and DT adjustment* ($P_{d}^{NUDT})$ *3: **ON INITIALIZED**: MIMO-OFDM signal (MIMO_OFDM_M×r*) *do:* ***Step 1****: Execution of simulation indicating detection probability impacted by DT adjustments* ($P_{d}^{DT}$) *and Detection probability impacted by DT adjustments and NU* ($P_{d}^{NUDT}$) vs. *SNR using* (*14*) (*22*)*,* (*28*)*, and* (*34*) *4: set pp = number of Monte Carlo simulations* *5: set SNR = signal to noise ratio in interval [−25 dB, 25 dB]* *6: **FOR** b = 1:length* (*SNR*) *7:*  *j1 = 0; j2 = 0;* *8: **FOR***
*pp = 1:10,000;* ***Step 2:***
*Modeling AWGN noise with varince*
$\sigma_{w}^{2}\left(n\right)$ *9: Noise_DT (ρ = 1.00,*
$\rho^{\prime }\;$*> 1.00*) *= sqrt*($\sigma_{w}^{2}(n)=1.00$).*randn (*1, mimo_ofdm_len*)*;* *10: Noise_NUDT* (*ρ > 1.00,*
$\rho^{\prime }\;$*> 1.00*) *= sqrt*($\sigma_{w}^{2}(n)>1.00$).*randn (*1, mimo_ofdm_len*)*;* ***Step 3:*** *Estimation of received signal*
y(*t*) *11:  finall_OFDM_M×r_DT*
*= MIMO_OFDM_M×r*
*+*
*Noise_DT;* *12:  finall_OFDM_M×r_NUDT*
*= MIMO_OFDM_M×r*
*+*
*Noise_NUDT;* ***Step 4:***
*Energy estimation of received signal using SLC concept* *13: **REPEATE FOR r= 1:R*** *14:  energy_calculation_DT*
*= abs*(*finall_OFDM_M×r_DT*)*.^2;* *15:  energy_calculation*
*_NUDT= abs*(*finall_OFDM_M×r_NUDT*)*.^2;* *16: **END*** ***Step 5:*** *Estimation of test statistics based on mixing energies of R signals*
*using* (*4*) *17: **FOR r= 1:R*** *18:  test_statistc_DT = sum*(*energy_calculation_DT*)*;* *19:  test_statistic_NUDT =*
*sum*(*energy_calculation_NUDT*)*;* *20: **END*** ***Step 6:***
*Threshold estimation using* (*18*)*,* (*20*)*, and* (*30*)*,* (*32*)) *21:  threshold_DT* (*b*) *=* ((*qfuncinv*($P_{fa}$(*b*))*./sqrt*(*N*))*+ 1*)*./*$\rho^{\prime }$*;* *22:  threshold_NUDT* (*b*) *=* ((*qfuncinv*($P_{fa}$ (*b*)).* ρ./sqrt(*N*))*+ ρ*)*./*ρ′*;* ***Step 7:***
*Making a final decision by using using* (*5*) *and* (*6*) *23: **IF*** (*test_statistc_DT >= threshold_DT* (*b*))*;* *24:  j1 = j1 + 1;* *25: **END*** *26: **IF*** (*test_statistic_NUDT >= threshold_NUDT* (*b*))*;* *27:  j2 = j2 + 1;* *28: **END***
*29: **END*** ***Step 8:***
*Evaluation*
$P_{d}^{DT}$
*and*
$P_{d}^{NUDT}$
*using Monte Carlo simulation* (*based on* (*3*)) *30:*  $P_{d}$*_DT* (*b*) *= i1/pp;* *31:*  $P_{d}$*_NUDT* (*b*) *= i2/pp;* *32: **END*** *33: **UNTIL***
$P_{d}^{DT}$*,*$\;P_{d}^{NUDT}$ *= [0, 1]*

The pseudocode of the developed algorithm is shown in Algorithm 1. To obtain the statistical relevance of the simulation results for different ED operation scenarios, the Monte Carlo simulations were executed according to Algorithm 1.

### Execution Steps of the Simulation Algorithm

The input parameters used in the performance evaluation of the ED method employing SLC technique are set in the first line of Algorithm 1. The parameters are the noise variance ($\sigma_{w}^{2}$), the received MIMO-OFDM signal (*MIMO_OFDM_M×r*), the overall number of sampling points (*N*), the length of the MIMO-OFDM data (*mimo_ofdm_len*), the false alarm probabilities ($P_{fa}$), the Monte Carlo simulation number (*pp*), the range of simulated SNRs (*SNR*), and the NU (ρ) and DDT factor ($\rho^{\prime }$).

The MIMO-OFDM signal (*MIMO_OFDM_M×r*) represents the signal sensed at SU antennas of the *R* Rx chains. This signal is generated for the different modulation types, PU Tx powers, the number of SU Rx and PU Tx chains (antennas), and the number of sampling points used in the ED. In simulated MIMO-OFDM CR systems, the received signal (*MIMO_OFDM_M×r*) is used as an input signal for the evaluation of the ED performance employing the SLC method. In Table 3, the exact values of the parameters used in the simulations are presented. 

In Algorithm 1 lines 4–8, the overall number of Monte-Carlo simulations is set. The Monte Carlo simulations are executed for a span of different SNRs (Table 3). In lines 9–10 (Step 2 of Algorithm 1), the AWGN noise with a mean value equal to zero and a variance of $\sigma_{w}^{2}$ is generated. The chosen values of noise variance are typical of those in practical wireless environments (Table 3).

Lines 11–12 (Step 3) represent the ED of two types of sensed signal during the ED process employing the SLC technique. The first signal (*finall_OFDM_M×r_DT*) is the MIMO-OFDM signal detected with DT adjustments ($\rho^{\prime }$ *>* 1.00) and without the impact of noise variations (*ρ =* 1.00) on the ED process. The second received signal (*finall_OFDM_M×r_NUDT*) is the MIMO-OFDM signal detected with the implementation of DT adjustments ($\rho^{\prime }\;$*>* 1.00) and the impact of NU (*ρ >* 1.00) on the ED process.

In lines 13–15 (Step 4), the calculation of signal energy after the SLC of the signals for each type of sensed signal (*energy_calculation_DT* and *energy_calculation_NUDT*) is performed. The tested statistic (energy) estimation for the signals sensed at the *R* Rx chains (antennas) of SU is shown in lines 17–20 (Step 5). In Algorithm 1, two cases of test statistic calculated according to Equation (4) are shown. The first one is the test statistic executed for the OFDM signals sensed without the impact of NU and with the DT adjustments (*test_statistc_DT*). In the second case, the signals are sensed for the simulation of the operating environment in which the impact of NU and with DT adjustments is considered during the ED (test_statistc_NUDT).

Algorithm 1 lines 21–22 (Step 6) represent two analyzed cases of the DT evaluation. In the first case (*threshold_DT* (*b*)), the DT evaluation is performed without the impact of NU and with DT adjustments. In the second case (*threshold_NUDT* (*b*))*,* the impact of both, the NU and ED exploiting the DT adjustments is considered. The mathematical expression for these two cases is presented in Equations (18), (20), (30) and (32), respectively.

Finally, the decision-making process which results in cognition about the exploitation of the frequency band by the SU is executed in lines 23–29 (Step 7). The decision-making process is performed based on testing the binary hypothesis presented by Equation (3). If the energy of the sensed signal is greater or the same as the set threshold, hypothesis $H_{1}$ is confirmed and the PU signal is present. Alternatively, if the energy is lower than a set DT, hypothesis $H_{0}$ points to an absence of PU and the presence of a spectrum hole. 

In lines 30–33 (Step 8), a large number of repeated Monte Carlo simulations were executed for the different SNR ranges and for every simulation environment of the ED. 

## 5. Results of Simulations

In Section 5, the simulation software used for performing comprehensive simulations for different ED operating environments is presented and the obtained simulation results have been thoroughly discussed. The process of SS was analyzed through the performance of the ED method based on the SLC technique in SISO and both asymmetric and symmetric MIMO-OFDM CR systems. The effect of NU on the transmitted MIMO-OFDM signal was modeled through simulations performed for the different NU factors. The process of signal detection at the location of SU was performed for various levels of the DT adjustment (DDT factors). The presented results analyze how different Tx-Rx antenna combinations at PU and SU sides, OFDM modulation constellations, the number of sampling points, Tx power levels, and SNRs influence the detection probability of the PU signal during the SS performed using the ED method employing the SLC technique. 

### 5.1. Description of the Simulation Parameters and Software

For the simulation of the ED in different operating environments mathematically formulated in Section 3, the Matlab 2016 software was used. The values of all simulation parameters are presented in Table 3. The simulations were performed for the transmission of OFDM signals modulated using the most practically applied modulation schemes such as 16/64 quadrature amplitude modulation (16/64—QAM) and quadrature phase shift keying (QPSK). In addition, the ED performance was analyzed for the number of sampling points used in the ED equal to 128, 512, and 1024 (Table 3). Furthermore, the analysis examined the SNRs ranging between −25 dB and 25 dB in the position of SU. The values of SNR in this SNR range are being frequently experienced in real practical environments as SNRs of many communication systems based on OFDM. To improve the reliability and accuracy of the simulations, 10,000 Monte Carlo simulations per each simulation run were executed (Table 3). This number of Monte Carlo simulations were selected for analysis for the purpose of achieving balance among the simulation durations and simulation accuracies. This enables the execution of the simulation algorithm for all simulated ED operation environments in the time frame of a few tens of microseconds. 

To analyze the impact of versatile levels of NU and DT adjustments on the performance of ED employing SLC principles, different combinations of values of DDT and NU factors have been selected for analysis (Table 3). More specifically, values of DDT and NU factors equal to 1.03 and 1.05 represent the lower and higher levels of DT adjustments and NU in the simulation analysis, respectively. To exclude the influence of DT adjustments and NU from the simulation analysis, DDT and NU factors equal to one ($\rho^{\prime }\;$= ρ = 1.00) were used in the simulation analyses.

### 5.2. Effect of the Number of Transmit Chains on ED Efficiency

**Figure 2:**
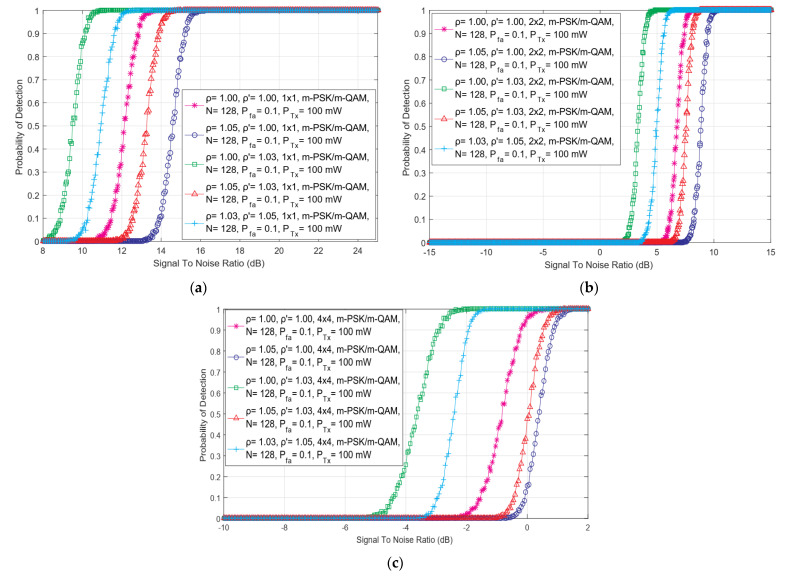
The dependency of detection probability on SNR for ED performed with different combinations of DDT and NU factors in (**a**) SISO, (**b**) symmetric 2 × 2 MIMO and (**c**) symmetric 4 × 4 MIMO communication systems.

**Figure 3:**
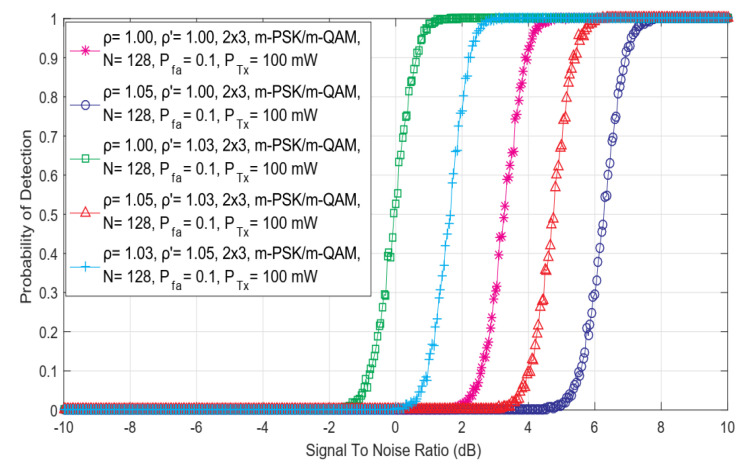
The dependency of detection probability on SNR for ED performed with different combinations of DDT and NU factors in asymmetric 2 × 3 MIMO communication system.

The simulation results shown in this section were used to analyze the impact of SISO, symmetric (Figure 2) and asymmetric (Figure 3) MIMO transmissions on the ED performance. Simulation results were obtained for the constant values of PU Tx power (*P =* 100 mW), the fixed number of sampling points used for the ED (*N* = 128), and the predefined false alarm ($P_{fa}$) probabilities equal to 0.1. The same results have been obtained independently of the used OFDM modulation schemes (64 QAM, 16 QAM and QPSK) and in Figure 2 and Figure 3, this is denoted by the m-PSK/m- QAM notation. For each Tx-Rx diversity schema, the assessment of the ED performance for diverse levels of DDT (ρ′) and NU (ρ) factors was performed. 

In Figure 2, the relationship among the SNR and detection probability for different combinations of DDT and NU factors and versatile symmetric MIMO communications systems (such as 1 × 1, 2 × 2 and 4 × 4) has been presented. Similar results have been presented in Figure 3 for the 2 × 3 asymmetric MIMO communications system. The results presented in Figure 2 and Figure 3 indicate that for higher values of SNR, the detection probability will be better for any symmetric or asymmetric MIMO Tx-Rx diversity combination and vice versa. Since the performance of the ED method is poor for lower values of SNR, a better detection probability can be achieved in the case of communication systems with a higher number of Rx and Tx chains on the PU and SU side, respectively. In addition, results in Figure 2 show that for the equal levels of SNRs, higher detection probabilities of PU signal will be achieved for Tx-Rx diversity combinations with a higher number of SU Rx and PU Tx chains. This improvement in detection probability is a consequence of the transmission diversity which enhances the precision of PU signal detection in the case where the larger numbers of PU Tx and SU Rx chains (antennas) will be used in the MIMO communication systems. 

### 5.3. Effect of NU and DT Adjustments on ED Efficiency

The results shown in Figure 3 further indicate the absence of the impact of the OFDM modulation scheme on the probability of detection ($P_{d}$). More specifically, for the same operating environment simulating the ED performance, the probability of detection is the same for any of the *m*-QAM or *m*-PSK constellations. These results indicate that the OFDM modulation type does not have an effect on the detection probability in the ED for any combination of the Tx-Rx chains at any SNR level (this can be also confirmed by Equations (11), (14), (17), (22), (23), (28), (29) and (34)). This is due to the dynamic adjustments of the OFDM modulation of the signal transmitted at a constant Tx power in rate-adaptive OFDM systems. In such transmission systems, at the location of SU, the signal is affected by the fluctuations in noise power (NU). According to this fact, the analysis further showed a strong influence of the NU and DT adjustments on the ED.

As shown in Figure 2 and Figure 3, for the same channel conditions, the performance of the ED will be degraded when SS is performed in operating environments with a stronger NU and with a fixed DT lacking any DT adjustments (ρ  = 1.05 and $\rho^{\prime }$ = 1.00). The results presented in Figure 2 and Figure 3 indicate that the ED will achieve a better probability of detection for the same SNRs when a higher level of DT adjustments according to the NU (e.g., ρ = 1.03, $\rho^{\prime }$ = 1.05 compared to ρ = 1.05, $\rho^{\prime }\;$= 1.03) will be performed during the ED (Figure 2 and Figure 3). A better probability of detection will be obtained if ED is performed in operating environments characterized by a lower level of NU and a higher level of DT adjustment (ρ = 1.03, $\rho^{\prime }$ = 1.05) and vice versa (ρ = 1.05, $\rho^{\prime }$ = 1.03). The best detection performance was obtained by the operating environments characterized by the lack of any NU and by the implemented DT adjustments during the ED ($\rho =1.00,\;\rho^{\prime }=1.03$). This operation scenario simulates the ED performed in the systems impacted by noise, which do not have fluctuations in time. This means that such fluctuations do not impact the ED. However, these operation scenarios are the least realistic since in real wireless communication systems, the noise from different sources and their power fluctuations (NU) occur as a frequent phenomenon. 

Hence, for all modulations schemas, a better detection probability can be reached only if the ED is based on DT adjustment ($\rho^{\prime }>1.00$). However, setting the DT adjustment at a too high or too low level can result in exceptionally high or exceptionally low DTs. This can cause high misdetections of the PU signal or a high sensitivity of ED. In both cases, the ED performance will be degraded. Therefore, the DT adjustments must be performed in accordance with the level of NU. This means that higher values of NU variation need to be followed by a greater level of DT adjustment and vice versa. 

### 5.4. Effect of the Transmit Power of PU on ED Sensing Efficiency 

In this section, the presented results of the simulation explain the effect of the diverse PU Tx powers on the detection performance in the SISO and symmetric MIMO (2 × 2, 4 × 4) wireless communication systems. The results were obtained for the ED operating environment characterized by OFDM signals transmitted with QPSK modulation and sensed with a permanent number of sampling points (*N* = 128), the fixed false alarm probability ($P_{fa})$ of 0.1 and the different levels of DT adjustments and NU levels.

**Figure 4:**
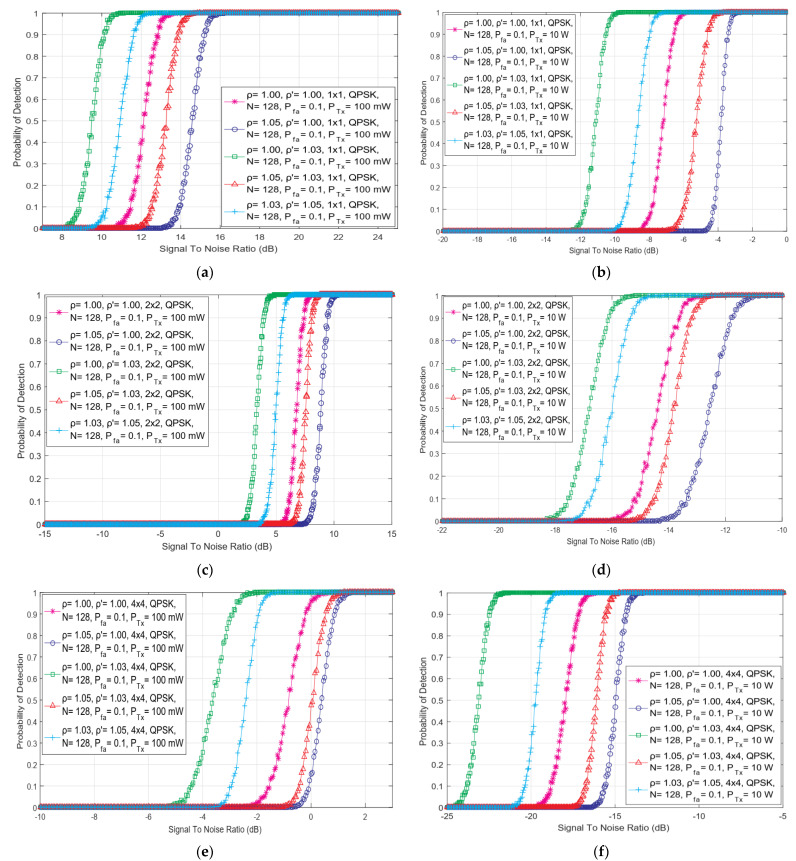
The dependency of detection probability on SNR of ED performed with different combinations of DDT and NU factors in communication: (**a**) SISO systems with 100 mW PU Tx power, (**b**) SISO systems with 10 W PU Tx power, (**c**) 2 × 2 MIMO systems with PU Tx power, (**d**) 2 × 2 MIMO systems with 10 W PU Tx power, (**e**) 4 × 4 MIMO systems with 100 mW PU Tx power and (**f**) 4 × 4 MIMO systems with 10 W PU Tx power.

In Figure 4, the dependency of detection probability on SNR for ED performed with versatile combinations of DDT and NU factors in SISO and MIMO communication systems, has been analyzed for two Tx powers of PU equal to 100 mW and 10 W. The obtained results shown in Figure 4 indicate that in SS environments having equal SNR levels at the antennas of SU, a higher detection probability will be for the PU signals transmitted at higher Tx powers. The transmission at Tx powers having higher values consequently results in a higher amount of the PU signal energy that will be eventually sensed at the Rx Chains of SU through the process of SLC. Additionally in Figure 4, it can be seen that the results of simulation show that the transmission of OFDM signal, which combines a larger number of Tx-Rx chains (4 × 4) and larger PU Tx power (10 W), has a positive effect on ED performance. As the number of Tx-Rx chains and PU TX powers increase, the simulation results shown in Figure 4 present that better probabilities of detection can be achieved for lower SNR values. Hence, the transmission of PU at higher Tx powers and in communication systems with a higher number of Tx-Rx chains, will always have a positive effect on the performance of the ED employing the SLC method. In addition, in ED operating environments characterized with lower values of SNRs at the Rx antennas of SU, performing transmission of MIMO-OFDM signal with a higher number of Tx-Rx chains and a higher PU Tx power, yield to the enhancement of the probability of PU signal detection in the SS process based on the ED method.

### 5.5. Effect of Differences in the Number of MIMO Tx and Rx Chains on the ED Performance

The results presented in this section show the analysis of the impact of the differences in the number of SU Rx and PU Tx chains in relation to the ED performance in SISO and symmetric MIMO systems. The results were obtained for ED realized in the operating environment characterized by the transmission of QPSK-modulated MIMO-OFDM signal, a constant number of sampling points (*N* = 128), constant PU Tx power (*P* = 100 mW), and targeted false alarm probability equal to 1.01.

**Figure 5:**
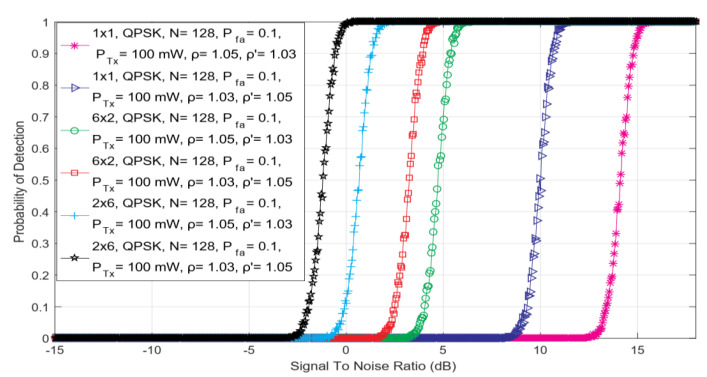
The dependency of detection probability on SNR for ED performed with versatile combinations of DDT and NU factors in SISO and asymmetric communication MIMO 2 × 6 and 6 × 2 systems.

In Figure 5, the dependency of detection probability on SNR for ED performed in SISO and asymmetric MIMO 2 × 6 and 6 × 2 communication systems have been presented. The results shown in Figure 5 point that the differences among the number of MIMO Tx and Rx chains impact ED performance. More specifically, the MIMO communication system having a higher number of Rx chains, will achieve higher detection probabilities for the PU signal at the same level of SNRs in the position of SU.

**Figure 6:**
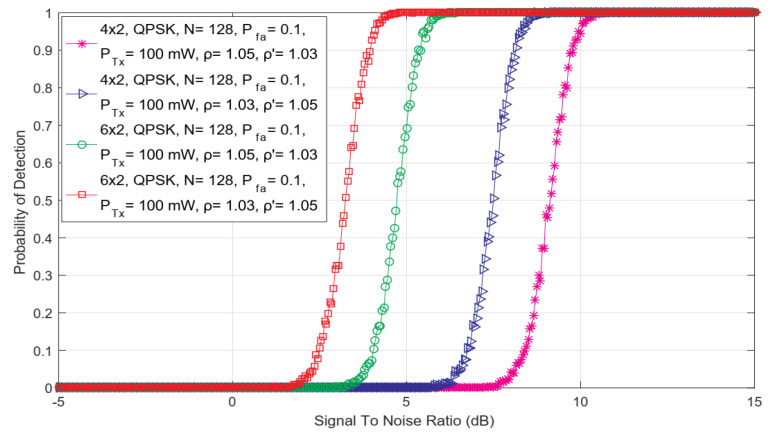
The dependency of detection probability on SNR for ED performed with different combinations of DDT and NU factors in asymmetric communication MIMO 4 × 6 and 6 × 4 systems.

These results are also confirmed in Figure 6, showing the dependency of the probability of detection on SNR for ED performed in asymmetric MIMO 4 × 6 and 6 × 4 communication systems. According to the results presented in Figure 6, for the same SNRs at SU antennas, the 4 × 6 MIMO communication system will achieve higher detection probabilities than the 6 × 4 communication system. Therefore, performing ED with SU having a larger number of Rx chains will contribute to the enhancement of ED performance. The main reason for this observation can be found in the fact that a larger number of Rx chains ensure the detection of more of the total energy of the PU signal in the process of SLC.

Additionally, the results presented in Figure 5 and Figure 6 indicate that the number of PU Tx chains also influences ED performance. The comparison of the results presented in Figure 5 and Figure 6 show that for the fixed number of Rx chains (antennas), a larger number of PU Tx chains will improve the probability of detection in MIMO-OFDM CR systems (better detection probability is for 6 × 2 in comparison with 4 × 2 systems). Therefore, it is to be expected that the ED performance in terms of sensitivity and accuracy of PU detection will be improved for the new generation of user and network devices which will have more transmission chains and corresponding antennas.

### 5.6. Effect of the Number of Sampling Points on ed Efficiency

The impact of the various number of sampling points used in the ED based on the SLC technique in SISO and asymmetric MIMO systems (2 × 2 and 4 × 4) has been analyzed in this section. The analysis was performed for the ED operating environment characterized by the transmission of a QPSK-modulated PU signal, the constant PU Tx power (*P =* 100 mW) and the constant values of false alarm probability ($P_{fa}=$0.1).

**Figure 7:**
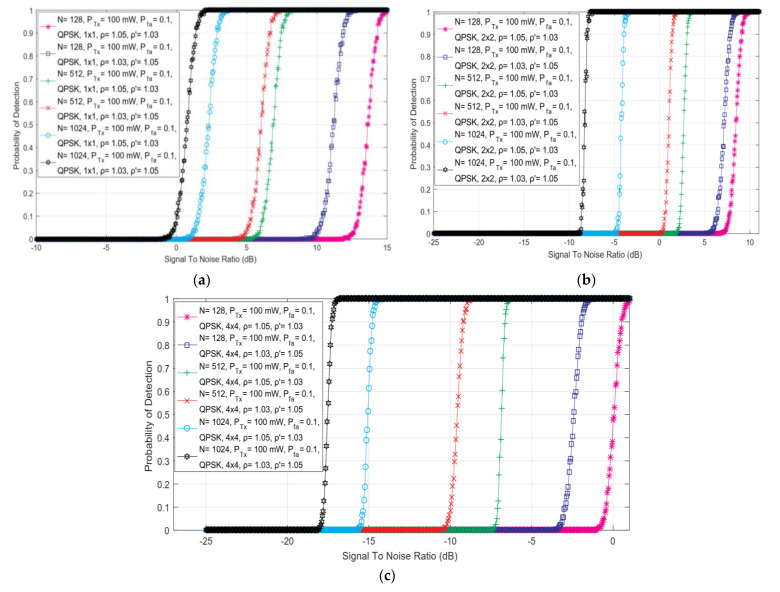
The dependency of the probability of detection on SNR for ED performed with the versatile number of sampling points and combinations of DDT and NU factors in (**a**) SISO, (**b**) 2 × 2 symmetric MIMO and (**c**) 4 × 4 symmetric MIMO communication systems.

In Figure 7, the dependency of detection probability on SNR for ED performed with versatile numbers of samples and combinations of DDT and NU factors in SISO and symmetric MIMO 2 × 2 and 4 × 4 communication systems has been presented. The obtained results indicate that for any combination of MIMO Tx-Rx chains and NU/DDT factors, the detection probability will be improved with an enlargement of the number of sampling points used for performing ED employing the SLC method. This is also confirmed in Equations (13), (21), (27) and (33), showing that ED performed with a larger number of sampling points *N* results in higher detection probabilities for the PU signal.

This is due to the fact that ED performed with a larger number of sampling points in practice means performing the PU signal sensing with a higher number of sensing attempts during the ED, which increases the probability of PU signal detection.

Additionally, the results presented in Figure 2, Figure 3, Figure 4, Figure 5 and Figure 6 including Figure 7 indicate that there exists an SNR-wall below which the detection probability cannot be ensured ($P_{d}\;$= 0). According to Figure 7, the levels of the SNR-walls shrink towards lower values of SNRs for ED performed in MIMO communication systems which have a larger number of Tx-Rx chains and for ED performed by the SU exploiting a higher number of sampling points. For example, the SNR-wall for a SISO system (1x1) performing ED with *N* = 128 samples (Figure 7a) will be significantly lower than the SNR-wall in symmetric MIMO 4 × 4 system performing ED with *N* = 1028 samples (Figure 7c). Consequently, the trade-off between the number of sampling points and the number of Tx-Rx chains on the PU and SU sides used in the ED, can have a significant influence on the probability of detection.

Furthermore, the results presented in Figure 7 were obtained for a different combination of NU and DDT factors ($\rho =1.05,\;\rho^{\prime }=1.03\;\mathrm{and}\;\rho =1.03,\;\rho^{\prime }=1.05$). The results indicate that for the same number of sampling points used in the ED, a better probability of detection will be obtained for the systems with a higher capability of DT adjustments ($\rho^{\prime }\;$=1.05) during the ED impacted by moderate NU (ρ = 1.03), than the systems with the lower capability of DT adjustments ($\rho^{\prime }$ = 1.03) which are impacted by a high NU (ρ = 1.05). This is the consequence of the fact that lower levels of NU (factor) have a lower negative impact on ED performance. This can be compensated by the exploitation of the increased number of sampling points in the ED, which contributes to improving the detection probability of the PU signals. On the other hand, in ED operating environments impacted by large NU, an assertion of NU must be followed by an appropriate (dynamic) adjustment of the level of DT during the ED process. Therefore, the trade-off within the number of sampling points used for the ED, the appropriate DT adjustment during the ED and the number of SU Rx and PU Tx chains, strongly impact the effectiveness of the ED at the location of SU. 

### 5.7. Effect of Probabilities of a False Alarm on the Efficiency of the ED Operation

In this section, the analysis of the results of simulations showing the effect of different levels of false alarm probabilities on detection probability in SISO and symmetric 4 × 4 MIMO-OFDM CR systems have been presented. The analysis was performed for the two different values of false alarm probabilities (0.01 and 0.2), the constant Tx power of PU (100 mW), the number of sampling points equal to 128, the QPSK modulation of PU signal, and the different levels of DT adjustments and NUs (ρ = 1.05, $\rho^{\prime }$ = 1.03 and ρ = 1.03, $\rho^{\prime }$ = 1.05). 

**Figure 8:**
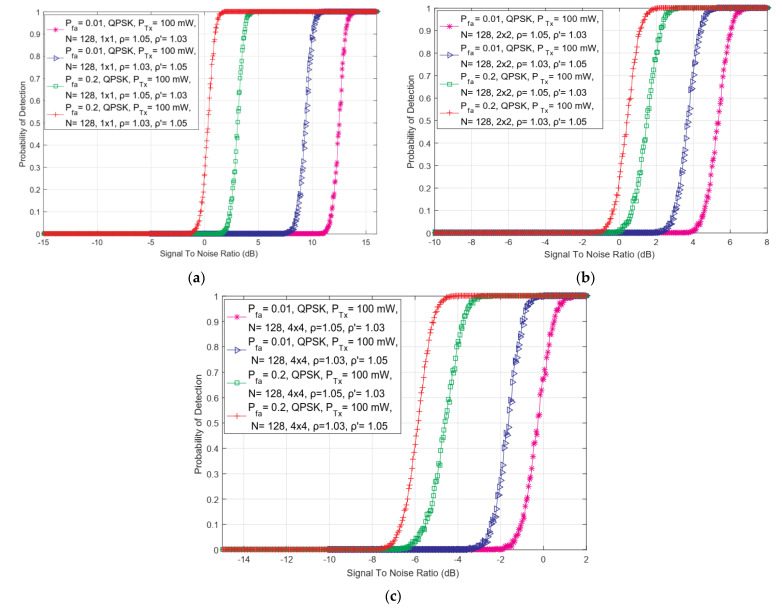
The dependency of detection probability on SNR for ED performed with different false alarm probabilities and combinations of DDT and NU factors in (**a**) SISO, (**b**) 2 × 2 symmetric MIMO and (**c**) 4 × 4 symmetric MIMO communication systems.

In Figure 8, the dependency of detection probability on SNR for ED performed with distinct false alarm probabilities and combinations of DDT and NU factors in SISO and symmetric 2 × 2 and 4 × 4 MIMO communication systems have been presented. The results indicate that for the same SNR level, the detection probability becomes lower as the values of targeted false alarm probability ($P_{fa}$) decrease and vice versa. These trends are characteristic for both SISO and symmetric MIMO systems and for all analyzed combinations of NU and DDT factors. These results are a consequence of the fact that when there is no exploitation of spectrum by PU, the probability that the SUs incorrectly estimate that the transmission of PU exists increases.

As in the case of the simulation results presented in Figure 2, Figure 3, Figure 4, Figure 5, Figure 6 and Figure 7, Figure 8 also shows that higher values of the DT factor ($\rho^{\prime }$ = 1.05) improve the detection probability for the equal false alarm probability ($P_{fa}$) at specific SNRs detected at the antennas of SU Rx chains. This cognition points to the importance of the implementation of appropriate DT adjustments in the ED process, for any targeted value of false alarm probability ($P_{fa}$) characteristic for ED based on CFAR principles. Furthermore, from Figure 8, it can be seen how the combination of the larger number of Tx-Rx chains and the higher values of requested false alarm probability ($P_{fa}),$ lead to a better detection probability for the same SNR values at the position of SU. On the other hand, a higher false alarm probability can result in an incorrect decision being made about PU activity in the ED process performed by SU. For that reason, an appropriate selection of minimal targeted values of false alarm probability for a specific number of Tx-Rx chains should be considered in the case of any operating environments in which the ED occurs. 

## 6. Conclusions

In this paper, the performance of the ED SS employing the SLC technique with DT adjustments according to NU variances in MIMO-OFDM CR systems has been analyzed. The mathematical expression of the main parameters used for the evaluation of the ED performance as a local SS technique employing SLC in MIMO-OFDM CR stems has been introduced. In addition, the algorithm for simulating the ED in versatile operating environments characterized by the influence of distinct levels of NU and performed with DT adjustments has been presented. The analysis of ED sensing efficiency has been performed through extensive simulations which indicates how different working parameters including the number of sampling points used in the ED, the Tx powers of PU, the DDT and NU factors, the probabilities of false alarm, and the SNRs impact the probability of the detection of PU signals in MIMO-OFDM CRs systems. 

The results of the analysis reveal that a general improvement in the SS efficiency of the ED method employing the SLC technique can be achieved if the level of DT adjustments during the ED follows the intensity of the variances in NU. The analysis also shows that higher values for the Tx power of PU, the number of sampling points, the false alarm probability, and the number of SU Rx and PU Tx chains can positively impact ED performance. Exploiting the cognitions shown in this paper in terms of selecting appropriate operational parameters such as the Tx power levels of PU, the number of sampling points, the number of Tx-Rx chains at PU and SU side and the expected false alarm probabilities can enable the implementation of more efficient ED SS in MIMO-OFDM CR systems. 

The obtained results further show that ED employing SLC in MIMO-OFDM CR systems containing multiple PU Tx and multiple SU Rx chains, outperforms in terms of ED efficiency the SISO communication systems. Additionally, in an asymmetric MIMO-OFDM CR system, configurations with a number of SU Rx chains larger than the number of PU TX chains yield better results with respect to ED performance of communication systems having an opposite ratio of Tx and Rx chains. Therefore, the proliferation of new user and network devices containing a higher number of build-in transmission chains will enable the improvement of ED performance in its practical implementation. Our future research work will be pointed towards the analysis of how massive-MIMO communications in terms of a large number of SU Rx and PU Tx chains in multi-cell CR networks, impact the performance of ED SS employing the SLC technique.

## Figures and Tables

**Table 1:** Literature survey of related work.

Reference	Major Contribution
[18]	Improved SS at the SU side in a realistic environment by employing SLC and square-law selection (SLS) techniques.
[19]	Software radio implementation of MIMO-OFDM.
[20]	A comprehensive survey of OFDM transmission for wireless communications.
[21]	A detailed survey on the performance requirements of 5G wireless cellular communication systems in terms of capacity, data rate, spectral efficiency, latency, energy efficiency, and quality of service.
[22]	In comparison with single antenna CRs systems, significant improvement is observed in PU detection probability when ED based on the SLC technique is performed in MIMO CRs systems.
[23]	Multiple antenna techniques and cyclostationary feature detection-based systems are proposed for ED.
[24]	Analysis of cooperative spectrum sensing with ED over various fading channels using the SLC diversity scheme.
[25]	Analyses of the problem of ED of an unknown signal over a multipath channel by employing SLC and SLS techniques.
[26]	The tutorial presents a comprehensive overview of the ED-based SS and provides tools necessary for performing analyses of several SS algorithms.
[27]	A survey of the NU impact on ED in communication systems with different OFDM system designs has been presented.
[28]	A review of ED performance exploiting dynamic DT adaptations in the SISO-OFDM systems.
[29]	Presentation of a novel approach based on subchannel and transmission power allocation that adaptively assigns the radio resources considering the interference caused to the PUs in multi-cell wireless networks.
[30]	Analyses of the new communication approach based on the licensed shared access (LSA) spectrum sharing framework with in-band full-duplex multi-cell multi-user MIMO communication network as the licensee, which operates in the service region of a multi-user MIMO incumbent network.
[31]	Presentation of the simulation algorithm that enables the performance analysis of the ED method employing the SLC technique in MIMO-OFDM CR systems and analyses of simulation results.
[32]	Analyses of efficiency of ED SS - based on SLC technique in MIMO-OFDM Cognitive Radio Networks without the impact of NU and dynamic DT adjustments.
[33]	Presentation of novel transmission solution based on adaptive beamforming with the coding scheme based on STBCs in IEEE 802.11 n WLAN systems.
[34]	Presentation of the current state-of-the-art related to the research on SS by using ED with an extensive overview of basic theories in recent research, architectures for performing ED SS, the possible applications of ED and performance measurements of ED.
[35]	The analysis of optimal DT selection for SS in a CRN using the ED approach is performed for fixed detection and false alarm probabilities.
[36]	A survey of the fundamental concepts of CRN characteristics, functions, network architecture and applications is presented.
[37]	The introduction of the ED SS which reduces the SNR-wall problem caused by the NU effects through the cooperation of multiple receivers for adapting the DT at each sensing point to the noise power present at the moment of SS.
[38]	A new ED algorithm based on dynamic DT selection is presented and the relationship of detection sensitivity and ED performance with the impact of fluctuation of average noise power is investigated.
[39]	Analyses of the influence of DDT and NU factor in the case of ED SSs on the detection and false alarm probability with the significance of their ratio on the sensing technique is analyzed and the expression of the empirical relationship between the sampling number and SNR is also proposed.
[40]	Development of the analytical model for estimation of the statistical performance of the ED which can be used for setting the appropriate DT such that more spectrum sharing can be exploited, especially when combined with cooperative SS.

**Table 2:** Parameters used in the simulation analysis.

Index	Description
$H_{1}$	The hypothesis which defines the existence of the PU signal
$H_{0}$	The hypothesis which defines the non-existence of the PU signal
*m*	The number of Tx chains on the PU side
*r*	The number of Rx chains on the SU side
*M*	The total number of PU Tx chains
*R*	The total number of SU Rx chains
*N*	The overall number of sampling points utilized for ED without DT adjustment and influence of NU
$N^{DT}$	The overall number of sampling points utilized for ED with DT adjustment
$N^{NU}$	The overall number of sampling points utilized for ED influenced by NU
$N^{NUDT}$	The overall number of sampling points utilized for ED with DT adjustment and influence of NU
$s_{m}$	The complex signal carried via the *m*-th Tx chain of the PU
s	The complex signal of the PU transmitted over the *M* Tx chains
*P*	The total via *M* Tx chains transmitted instantaneous Tx power
$P_{m}$	Instantaneous Tx power transmitted on the PU *m*-th antenna chain
$y_{r}\left(n\right)$	Vector of the signal detected at *r*-th Rx chain of the SU in the *n*-th SS period
Y(n)	Vector of the signal received by all *R* Rx chains of the SU in the *n*-th SS period
$h_{r\;}\left(n\right)$	Vector of channel gain among the *M* Tx chains and the *r*-th Rx chain in the *n*-th SS period
$s_{r}\left(n\right)$	Vector of the signal detected within the *n*-th SS sample point at the SU *r*-th Tx chain
$w_{r}\left(n\right)$	Vector of the noise impacting ED during the *n*-th SS period at the *r*-th Rx chain of the SU
$\sigma_{w}^{2}{}_{r}\left(n\right)$	The variance of noise for the signal detected in *n*-th SS period at the SU *r*-th Rx chain
$\sigma_{s_{r}}^{2}\left(n\right)$	The variance of the received signal in the *n*-th SS period at the *r*-th Rx chain of the SU
$\sigma_{wNU}^{2}\left(n\right)$	AWGN variance used in the ED impacted with NU
$\sigma_{wNUDT}^{2}\left(n\right)$	AWGN variance used in the ED impacted with NU and DT adjustments
$\Lambda_{r}$	Test statistics for signals detected at the *r*-th Rx chain (antenna) of the SU
$\Lambda_{SLC}$	The overall test statistics of all signals detected via the *R* receive (Rx) chains of the SU
$\gamma_{r}\left(n\right)$	Signal-to-noise ratio at the *r*-th receive chain of the SU during the *n*-th SS period
$\gamma_{SLC}\left(n\right)$	The total signal-to-noise ratio associated with the *R* SU receive antennas (chains) in the *n*-th SS period
$\bar{\gamma_{SLC}}\left(n\right)$	The mean signal-to-noise ratio detected by the SU during the *n*-th SS period for all *R* receive chains
$P_{f}$	False alarm probability for ED performed without DT adjustments and impact of NU
$P_{d}$	Detection probability for ED performed without DT adjustments and impact of NU
$P_{fa}^{NU}$	False alarm probability for ED impacted with NU
$P_{d}^{NU}$	Detection probability for ED impacted with NU
$P_{fa}^{DT}$	False alarm probability for ED performed with DT adjustments
$P_{d}^{DT}$	Detection probability for performed with DT adjustments
$P_{fa}^{NUDT}$	False alarm probability for ED performed with DT adjustments and impact of NU
$P_{d}^{NUDT}$	Detection probability for ED performed with DT adjustments and impact of NU
Q(x)	Standard Gaussian Q function
λ	DT for ED performed without DT adjustments and impact of NU
$\lambda_{fa}$	False alarm threshold in the case of ED performed based on CFAR principles
$\lambda_{d}$	DT level for ED performed based on CDR principles
$\lambda_{d}^{DT}$	DT for SLC ED performed with DT adjustments
$\lambda_{fa}^{DT}$	False alarm threshold for ED performed with DT adjustments
$\lambda_{d}^{NU}$	DT for ED impacted with NU
$\lambda_{fa}^{NU}$	False alarm threshold for ED impacted with NU
$\lambda_{d}^{NUDT}$	DT for SLC ED performed with DT adjustments and impacted with NU
$\lambda_{fa}^{NUDT}$	False alarm threshold for ED performed with DT adjustments and impacted with NU
$\lambda^{\prime DT}$	DT for ED performed without NU
$\lambda^{\prime NUDT}$	DT for ED performed with DT adjustments and NU
*ρ*	NU factor
$\rho^{\prime }$	DDT factor

**Table 3:** Parameters used in the process of simulation.

Parameters	Type/Quantity
PU signal modulation scheme	OFDM
Number of Tx chains (antennas) of the PU	1–4
Number of Rx chains (antennas) of the SU	1–6
OFDM modulation schemes	64 QAM, 16 QAM, QPSK
Model of the noise [28,34]	AWGN
$\mathrm{Noise}\;\mathrm{variance}\;\sigma_{w}^{2}\;\mathrm{for}\;\mathrm{DT}\;(\rho =1.00,\;\rho^{\prime }>1.00)$ [37,38,39,40]	1.00
$\mathrm{Noise}\;\mathrm{variance}\;\sigma_{w}^{2}\;\mathrm{for}\;\mathrm{NU}\;\mathrm{and}\;\mathrm{DT}\;(\rho \;>1.00,\rho^{\prime }>1.00)$ [37,38,39,40]	1.01
Number of sampling points for ED (FFT size) [28,34]	128, 512, 1024
SNRs range at SU position (dB) [28,34]	−25–25
DT factor ρ′ [37,38,39,40]	1.00, 1.03, 1.05
NU factor ρ [37,38,39,40]	1.00, 1.03, 1.05
Target false alarm probability [37,38,39,40]	0.01, 0.2
Overall number of Monte Carlo simulations	10,000

## Abbreviations

The following abbreviations are used in this manuscript:

AWGN	Additive white Gaussian noise
BS	Base station
CFAR	Constant false alarm rate
CR	Cognitive radio
CRN	Cognitive radio networks
CSI	Channel state information
DSA	Dynamic spectrum access
DT	Detection threshold
DDT	Dynamic detection threshold
ED	Energy detection
EGC	Equal Gain Combining
IoT	Internet of Things
ISI	Inter-symbol interference
LSA	Licensed shared access
MIMO	Multiple-input multiple-output
MISO	Multiple-input single-output
NU	Noise uncertainty
OFDM	Orthogonal frequency-division multiplexing
PU	Primary user
RF	Radiofrequency
SISO	Single-input-single-output
SIMO	Single-input multiple-output
SL	Square-law
SLC	Square-law combining
SLS	Square-Law Selection
SNR	Signal-to-noise ratio
SS	Spectrum sensing
STBC	Space-time block codes
SU	Secondary users
5G	Fifth-generation mobile network
6G	Sixth-generation mobile network
